# The Experimental Autoimmune Encephalomyelitis Disease Course Is Modulated by Nicotine and Other Cigarette Smoke Components

**DOI:** 10.1371/journal.pone.0107979

**Published:** 2014-09-24

**Authors:** Zhen Gao, Jillian C. Nissen, Kyungmin Ji, Stella E. Tsirka

**Affiliations:** 1 Department of Pharmacological Sciences, School of Medicine, Stony Brook University, Stony Brook, New York, United States of America; 2 Program in Neuroscience, School of Medicine, Stony Brook University, Stony Brook, New York, United States of America; IDIBAPS, Spain

## Abstract

Epidemiological studies have reported that cigarette smoking increases the risk of developing multiple sclerosis (MS) and accelerates its progression. However, the molecular mechanisms underlying these effects remain unsettled. We have investigated here the effects of the nicotine and the non-nicotine components in cigarette smoke on MS using the experimental autoimmune encephalomyelitis (EAE) model, and have explored their underlying mechanism of action. Our results show that nicotine ameliorates the severity of EAE, as shown by reduced demyelination, increased body weight, and attenuated microglial activation. Nicotine administration after the development of EAE symptoms prevented further disease exacerbation, suggesting that it might be useful as an EAE/MS therapeutic. In contrast, the remaining components of cigarette smoke, delivered as cigarette smoke condensate (CSC), accelerated and increased adverse clinical symptoms during the early stages of EAE, and we identify a particular cigarette smoke compound, acrolein, as one of the potential mediators. We also show that the mechanisms underlying the opposing effects of nicotine and CSC on EAE are likely due to distinct effects on microglial viability, activation, and function.

## Introduction

Cigarette smoking has emerged as a major risk factor for multiple sclerosis (MS) [1], an autoimmune disease of the central nervous system (CNS) that affects over 2.5 million people worldwide (National Multiple Sclerosis Society). Compared with non-smoking MS patients, smokers develop more severe symptoms and have more aggressive secondary progression. A dose-response relationship exists for cigarette smoking and MS severity, and the incidence of MS increases with prolonged smoking exposure [2]–[4]. However, the mechanism by which cigarette smoking promotes MS remains unclear.

Nicotine has been suggested to contribute to cigarette smoking's detrimental effects in the context of MS. As nicotine increases microvascular blood flow [5] and the permeability of the blood brain barrier (BBB) [6], these effects could suffice to promote significant BBB leakage, an event important in the initiation of MS [7]. Nicotinic acetylcholine receptors (nAChRs) are expressed by immune cells that play critical roles in MS, including T cells [8], macrophages/microglia [9] and dendritic cells [10], raising the possibility that nicotine might stimulate immunomodulatory pathways that initiate or accelerate MS progression. However, recent studies suggested that nicotine did not promote more severe symptoms during EAE -, but rather inhibited disease development [11]. Moreover, evidence supported the idea that the use of tobacco moist snuff is not associated with increased MS risk [12], [13]. Since the use of moist snuff resulted in similar serum levels of nicotine to cigarette smoking, this suggested that components other than nicotine in cigarettes might actually underlie the adverse effects of cigarette smoking in MS.

Microglia are the resident macrophage-like immune cells in the CNS. They play critical roles during MS and its corresponding animal model (experimental autoimmune encephalomyelitis – EAE). Microglial activation is an early event in MS/EAE and persists throughout the course of the disease [14], [15]. MS/EAE is characterized by predominance of pro-inflammatory (M1) microglia, although both M1 and anti-inflammatory (M2) microglia modulate MS/EAE progression [16]. Inhibition of microglial activation by either the tripeptide macrophage/microglial inhibitory factor MIF (TKP) or minocycline ameliorates EAE symptoms [17]–[19]. Moreover, we have shown that shifting microglial activation towards an M2 state leads to a corresponding shift of T cells towards immunoprotective phenotypes, resulting in improved EAE outcomes [20]. Therefore, microglia have key roles in determining MS/EAE pathogenic outcomes, and pharmacological fine-tuning of their function could greatly affect disease progression.

The findings we described above were paradoxical in that cigarette smoking has been identified as a risk factor for MS, while nicotine has been described as having beneficial effects during the EAE disease course [1]–[4], [11]. We thus set out to examine whether the non-nicotine components of cigarette smoke underlay the linkage to MS/EAE development. In this study, we investigated and compared the roles of nicotine and non-nicotine components of cigarette smoke in EAE. Our results demonstrate that nicotine improves EAE symptoms, whereas cigarette smoke condensate (CSC), which contains the non-nicotine components of cigarette smoke but only 3% nicotine [21], worsens EAE severity during early stages of this model system, correlating with the presence of M1 microglia. These findings suggest that the non-nicotine components in cigarettes contribute to the detrimental effects to MS/EAE, while nicotine has strong potential as an MS therapeutic.

## Materials and Methods

### Animals

C57BL/6 (wild-type) mice were purchased from Jackson Laboratory and bred in-house under pathogen-free conditions on a 12-hour light/dark cycle. Access to food and water was *ad libitum*. All experimental procedures were in accordance to NIH guidelines and protocols approved by Stony Brook University Institutional Animal Care and Use Committee (IACUC) and the Division of Laboratory Animal Research.

### EAE induction and evaluation

EAE was actively induced by subcutaneous injection of MOG_35-55_ peptide (MEVGWYRSPFSRVVHLYRNGK) as previously described [17], [22]. Briefly, 8–10 week-old female wild-type mice were anesthetized with intra-peritoneal injection of atropine (0.6 mg/kg body weight) and 2.5% avertin (0.02 ml/g body weight). 300 µg MOG was emulsified in complete Freund's adjuvant (CFA, DIFCO) supplemented with 500 µg of heat-inactivated Mycobacterium tuberculosis (DIFCO) and injected into the mouse hind-flank on day 0. On day 7, mice were boosted with injection of 300 µg of MOG into the other flank. 500 ng pertussis toxin (List Biologicals) was injected intra-peritoneally on day 0 and 2. The MOG_35-55_ peptide was purchased from Quality Controlled Biochemicals, Inc and from the Yale University Peptide Synthesis Facility.

EAE clinical symptoms were scored blindly starting on day 3 on a scale of 0 to 5 with gradations of 0.5 for intermediate symptoms. 0, no detectable symptoms; 1, loss of tail tone; 2, hindlimb weakness or abnormal gait; 3, complete paralysis of the hindlimbs; 4, complete hindlimb paralysis with forelimb weakness or paralysis; 5, moribund or death. Weights were measured weekly starting from day 0.

### Quantification of Cotinine

The serum levels of cotinine, a metabolite of nicotine, were used as an indicator of nicotine levels in mice after nicotine and CSC delivery [23]. Blood samples were collected using sterile tubes without anticoagulants and left at room temperature for 30 minutes or until the blood was clotted. The tubes were then centrifuged at 3000 rpm for 10 minutes and the serum collected and stored at −80°C. Cotinine levels were measured using the Calbiotech cotinine direct ELISA kit following the manufacturer's protocol.

### Drug delivery

The amount and concentration of the nicotine solution administered *in vivo* was adapted from the literature (Shi et al., 2009). 200 mg/ml nicotine ditartrate solution in saline was freshly prepared and loaded into mini-osmotic pumps (14-day or 28-day, infusion rate of 0.25 µL/h, Alzet), as previously described (Shi et al., 2009). Pumps were incubated in saline at 37°C overnight (14-day pumps) or for at least 40 hours (28-day pumps) to ensure a steady pump rate (0.25 µl/hr) and then implanted subcutaneously in the back of the mice. At 14 or 28 days of infusion, the serum cotinine levels were measured at 83.8 ng/ml (14-day infusion) and 85.7 ng/ml (28-day infusion), which is comparable to that found in heavy smokers (80–100 ng/ml) [24]. The preparation of CSC is at 40 mg/ml in DMSO (Murty Pharmaceuticals). Since DMSO can be delivered effectively by osmotic pumps only when it is diluted in water/saline (up to 50%), the pump-delivered working concentrations of CSC were 20 mg/ml and 10 mg/ml for the two types of pumps. After 14 days of infusion, the serum cotinine levels in mice with CSC pumps (20 mg/ml) were measured at 1.54 ng/ml. Control mice received pumps containing vehicle (saline or DMSO). We compared clinical scores of DMSO- and saline-treated mice. There was no difference between these two groups (Figure S1). This result is in agreement with results described in the literature [25]. Depending on experimental design, the pumps were implanted either immediately after, 14 days after, or 14 days before the first injection of MOG.

### Tissue processing

Mice were deeply anesthetized with intra-peritoneal injection of 2.5% avertin (0.02 ml/g body weight) and then transcardially perfused using PBS (pH 7.4) followed by 4% paraformaldehyde (PFA)/PBS (pH 7.4). Spinal cords were isolated, post-fixed in 4% PFA/PBS at 4°C overnight, and dehydrated in 30% sucrose at 4°C until the samples sank. After the meninges were removed, the spinal cord was cut into equal sections, embedded in optimal cutting temperature compound (Tissue Tek), frozen and stored at −80°C until use.

### Immunofluorescence

Coronal sections (25 µm) were prepared with a cryostat (Leica). Spinal cord sections were blocked in 3% BSA in PBS-T (0.3% TritionX-100 in PBS) and incubated with primary antibodies (rabbit anti-Iba1 1∶500, Wako; mouse anti-iNOS 1∶500, BD Biosciences; mouse anti-Arg1 1∶500, BD Biosciences; rabbit anti-Caspase-3, 1∶1000, R&D systems; rat anti-Mac2 1∶1000, Cedarlane; rat anti-CD45, 1∶1000, BD systems) overnight at 4°C. Incubation with fluorescence-conjugated secondary antibody at 1∶1000 was performed for 1 hour at room temperature, followed by washing with PBS and mounting using Fluoromount-G with DAPI (Southern Biotech). 4–8 spinal cord sections in the lumbar region were imaged per biological replicate. In each section, 1–2 fields in the dorsal white matter and 5–6 fields in the ventral white matter (WM) were captured for analysis. ImageJ (NIH) was used to quantify the intensity of the fluorescent signals.

To quantify iNOS/Arg1 staining of spinal cords, images were taken at seven selected locations throughout the white matter that were consistent among all samples. For Iba1 intensity, images encompassing the entirety of a coronal section were taken, with seven images taken per animal. Values were quantified using ImageJ software by multiplying the staining intensity by the cell count, divided by the total tissue area. All quantification was non-blind.

For cell culture staining, primary microglia were seeded onto coverslips. After treatments, cells were stained using the protocol described above. Four fields selected at random per biological replicate were captured for imaging and quantification.

### Eriochrome cyanine staining and measure of demyelination

Slides were incubated with acetone for 5 min, air-dried at room temperature for 30 minutes, stained in eriochrome cyanine (EC) solution (0.2% eriochrome cyanine RS (Sigma), 0.5% H_2_SO_4_ (Sigma), 0.4% iron alum (Sigma) in distilled water) for 30 min, sequentially differentiated in 5% iron alum (Sigma) for 10 minutes and in borax-ferricyanide solution (1% borax (Sigma), 1.25% potassium ferricyanide (Sigma), in distilled water) for 5 min, dehydrated through graded ethanol solutions and mounted using Permount (Fisher Scientific).

6–9 spinal cord sections in the lumbar region were imaged per biological replicate. ImageJ (NIH) was used to measure the demyelinated and total areas of WM. The final % value for demyelination was calculated using equation listed below: 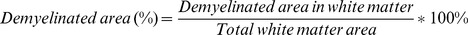



### Fluoromyelin staining

Slides were rehydrated in PBS for 5 minutes, incubated with fluromyelin staining solution (1∶300) for 20 minutes at room temperature, washed, and mounted with Fluoromount-G (Southern Biotech). The ImageJ freeware (NIH) was used to measure the demyelinated and total areas of WM as described above.

### Mixed cortical and primary microglia cultures

Mixed cortical cultures were prepared as previously described [26], [27]. Briefly, newborn pups (d0–d2) of wild-type mice were decapitated and the cortices freed from meninges, hippocampi and midbrain and kept in HBSS (Cellgro) on ice. The cortical tissue was digested in 0.25% Trypsin/EDTA (Cellgro) at 37°C for 20 min and triturated mechanically. After filtration, the cell suspension was plated in mixed cortical medium (DMEM, 10% FBS, 1mM sodium pyruvate, 40 µg/ml Gentamycin) onto poly-D-lysine coated tissue culture dishes (37°C, 5 µg/ml PDL). Medium was changed 3 days after plating.

Microglia were harvested 10 days after plating by adding lidocaine (15 mM, Sigma) into the medium for 15 minutes at room temperature. The medium containing the floating microglia was collected and centrifuged at 1500 rpm for 5 min. The cell pellet was then resuspended in an appropriate volume of microglia medium (DMEM, 1% FBS) and the cell number counted. The purity of the microglial cultures was>98% as described previously [26], [27]. The microglia were used for experiments after 24–48 hours of culture.

### Primary hippocampal neuronal culture

Mouse hippocampi of newborn pups (P0) were dissected, digested (0.25% trypsin in HBSS) at 37°C for 20 minutes and then triturated to form single cell suspensions. The cell suspension was filtered and the cells plated in neuronal medium (Neurobasal with B27 supplements, 0.5 mM L-glutamine) at a density of 100,000 cells/well on PDL-coated 12-well plates. AraC (10 µM) was added to the medium on day 2 of the culture to inhibit glial growth and the media replaced on day 4. After being cultured for 12 days, the neurons were treated with 100 µM glutamate for 24 hours and the medium collected and kept at −80°C until use.

### ELISA

Protein concentrations were measured using the Bio-Rad protein assay kit. Mouse TNF-α/IL-10 ELISA kit (eBioscience) was measured according to the manufacturer's instructions. In brief, a 96-well plate was coated overnight with capturing antibody. The antibody was then blocked with assay diluent for 1 hour at room temperature. 100 µl of samples or cytokine standards diluted in assay diluent were added and incubated for 2 hours at room temperature. Biotin-conjugated detection antibody, Avidin-HRP reagent, substrate solution and stop solution were then added into the wells sequentially and incubated for specific time-periods as per the protocol. Extensive washes were performed after each step. Absorbance at 450 nm was measured immediately by microplate reader after adding stop solution. Cytokine release was calculated using the equation below and the values normalized to neuronal conditioned medium (NCM)-treated samples. 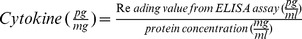



### Live/dead assay

Live/dead assay (Life Technologies) was conducted following manufacturer's instruction. Calcein AM (1∶2000) and ethidium homodimer-1 (1∶500) were added to the culture medium to label live and dead cells, respectively. Imaging was performed 10 minutes later; 4 fields were captured per biological replicate for quantification.

### Analysis

Results are presented as an average with error bars indicating the standard error of the mean (Mean ± SEM). Two-way ANOVA was performed for multiple comparisons in clinical score and weight changes results. For other experiments, two-tailed *t*-test was used for comparisons between two groups. One-way ANOVA was performed for comparisons between multiple groups. Significance is indicated by *p<0.05, **p<0.01, ***p<0.001.

## Results

### Non-nicotine components of cigarette smoke worsen EAE clinical symptoms, whereas nicotine attenuates the symptoms

**Figure 1:**
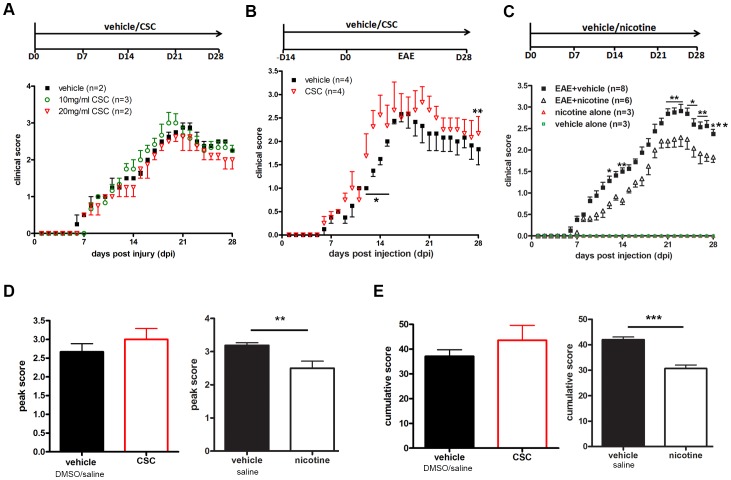
Effects of nicotine and non-nicotine components of cigarette smoke on EAE severity. EAE was induced by injection of MOG_35–55_ in CFA and pertussis toxin. **(A)** CSC was infused at 10 or 20 mg/ml into EAE mice starting at day 0 of EAE for 28 days, with DMSO as vehicle. **(B)** 20 mg/ml CSC was infused into the mice from 14 days prior to EAE induction through day 28, with 50% DMSO as vehicle. **(C)** Nicotine (200 mg/ml) was infused into EAE mice starting at Day 0 of EAE for 28 days, with saline as vehicle. Peak score **(D)** and cumulative score **(E)** were compared between CSC (n = 3)/nicotine (n = 6) and control mice (n = 8). (*** p<0.001; **p<0.01; *p<0.05).

To examine whether the non-nicotine components of cigarette smoke underlie the linkage to MS/EAE development, cigarette smoke condensate (CSC), a complex mixture of compounds that includes nicotine, was infused into mice using osmotic mini-pumps. The amount of nicotine in CSC is quite small, only approximately 2–3% of the total particulate matter (TPM). We first infused 10 or 20 mg/ml of CSC into EAE mice starting from day 0 of the protocol (the day when MOG was injected to initiate EAE) for 28 days. In the control (50% DMSO vehicle-treated) group, the clinical symptoms began around day 7, increased gradually, reached their peak between days 21–23, and then subsided (Fig. 1A). CSC infusion starting from day 0 resulted in similar EAE clinical symptoms (Fig. 1A). Since prior history as a smoker predisposes individuals to MS [4], we performed the experiment again using mice that were exposed to CSC for 14 days prior to MOG injection, in addition to infusion during the disease course. Using this paradigm, 20 mg/ml CSC significantly worsened EAE clinical scores during the early phase of the disease (day 12–15). For example, on day 13 the clinical score of CSC-treated mice was 2.31±0.69 as opposed to 1.38±0.25 for the vehicle-treated mice (Fig. 1B). However, it is possible that the effect of CSC on these animals was blunted by the presence of atropine used as pre-anesthetic. Atropine is a potent antagonist of muscarinic acetylcholine inhibitors, which have been shown to be expressed on a variety of immune cells. Further, studies have shown that atropine may have an effect on the immune system through suppressing inflammation [8]. Nevertheless, CSC application did not significantly change the peak or the cumulative score of EAE mice with the sample group sizes used in this experiment (Fig. 1D, E).

To assess nicotine's effect on EAE, a 200 mg/ml solution was infused into mice using osmotic mini-pumps. This dosage achieved steady-state serum levels of cotinine, a nicotine metabolite, at 83.8 ng/ml, which is comparable to the cotinine levels found in heavy smokers (80–100 ng/ml) [11], [24]. Drug delivery started on day 0 of the experiment and lasted for 28 days. Our results showed that the mice treated with nicotine became symptomatic with a similar temporal pattern as saline vehicle-treated mice, but exhibited a significantly milder course of disease (Fig. 1C). The effects of nicotine on mean EAE scores achieved significance on individual days during the early stages (days 11, 12, and 14, and 17), at the peak (day 21), and beyond (Fig. 1C). The average peak score for the control mice in nicotine experiments was 3.2±0.2, meaning that these mice exhibited total paralysis of hindlimbs accompanied by weakness of forelimbs (Fig. 1D). In contrast, the average peak score for the nicotine-treated mice was 2.5±0.5, equating to paralysis in one leg or severe weakness of both legs but without paralysis. More generally, the nicotine-treated EAE mice exhibited a significantly lower cumulative score (30.7±3.3) in comparison to the vehicle-treated EAE mice (42.0±2.9), indicating an overall attenuation of the disease severity (Fig. 1E). Infusion of either 5 mg/ml or 10 mg/ml nicotine via osmotic pump, a concentration higher than the one found in CSC (calculated at 0.48 mg/ml in the 20 mg/ml CSC dose) [28], had no effect on disease scores (Figure S2), suggesting that the effect of the nicotine present in CSC is negligible. Taken together, the non-nicotine components in CSC worsened EAE scores during early stages, while nicotine administration significantly attenuated EAE severity.

**Figure 2:**
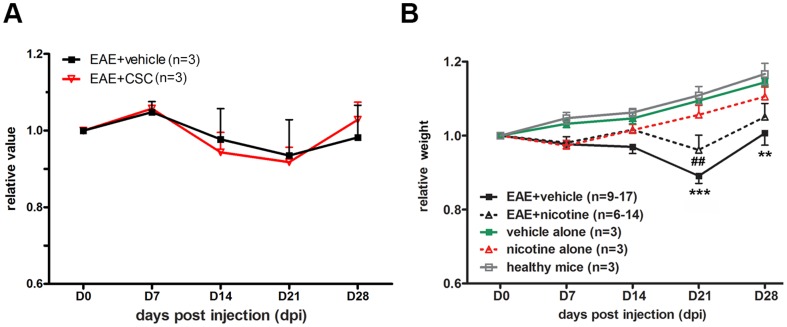
Weight changes of CSC- or nicotine-treated EAE animals. Weight changes were recorded and compared between vehicle (DMSO, n = 3 at all time points) and CSC- **(A)** or nicotine-treated **(B)** mice. In **(B)**, * denote significance compared to mice treated with vehicle (saline) alone (n = 3 at all time points). #: significance compared to mice treated with nicotine alone (n = 3 at all time points). Weights were plotted as a ratio over day 0 weights. (*** p<0.001; **p<0.01; ##p<0.01). On day 28 there were n = 9 mice in the EAE+vehicle group and n = 6 in the EAE+nicotine group. On day 21 there were n = 12 mice in the EAE+vehicle group and n = 11 in the EAE+nicotine group. On days 0, 7 and 14 there were n = 17 mice in the EAE+vehicle group and n = 14 in the EAE+nicotine group.

Weight loss is another important hallmark of MS patients and EAE animals. Body weights of the vehicle-treated EAE mice were measured weekly. As expected, we observed that, while control vehicle-injected non-EAE mice steadily increased in weight during the 28 days, the EAE mice lost weight relative to controls as they became strongly symptomatic, and then gained weight during the recovery phase (Fig. 2A, B). CSC-treated EAE mice did not show differences from vehicle-treated animals on weight change during the experiment (Fig. 2A).

### Nicotine and non-nicotine components have distinct effects on EAE-induced spinal cord demyelination

**Figure 3:**
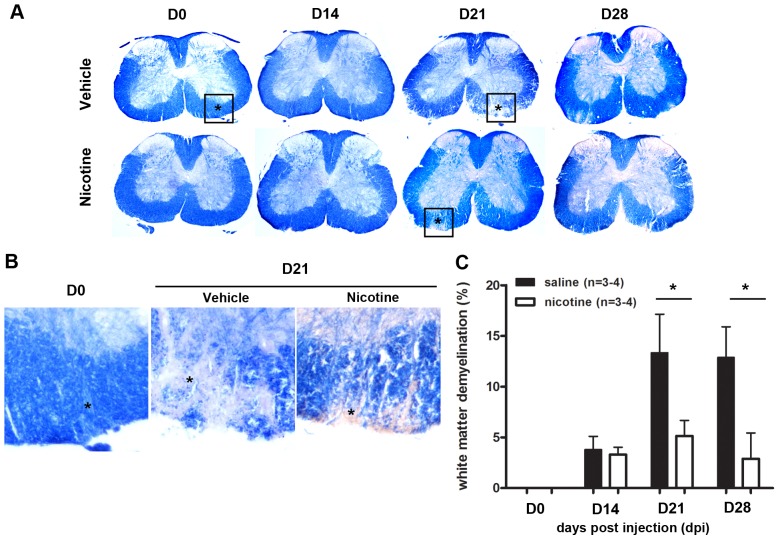
Decreased demyelination in nicotine-treated mice during EAE. **(A)** Frozen sections of spinal cords were isolated from vehicle (saline) and nicotine-treated mice at different time points during EAE. Eriochrome cyanine (EC) was used to visualize demyelination. Intact white matter (WM) is indicated by the blue staining and demyelination is revealed by diminished color. Areas of demyelination are indicated by asterisks; boxed regions are shown at higher magnification. **(B)** Demyelinated areas were measured using ImageJ and calculated based on equation listed in Methods. (n = 3–4, *p<0.05).

EAE is characterized by focal demyelination of the spinal cord, which can be assessed by staining spinal cord sections with fluoromyelin or eriochrome cyanine (EC), which adheres to myelin, creating either fluorescent or intense blue coloration; demyelination thus presents as sites of hypo-colorization. Spinal cords analyzed from vehicle-treated EAE mice displayed no areas of focal demyelination at day 0 (the day of injection of MOG to induce EAE) and only infrequent sites at day 14, but had many foci of demyelination at days 21 and 28 (Fig. 3A, B), correlating with the peak severity of symptoms. In contrast, the areas of focal demyelination were less frequent and smaller in the nicotine-treated EAE mice. Quantitatively, equivalent amounts of demyelination (about 3.3–3.8%) were observed in vehicle- and nicotine-treated EAE mice at day 14, which correlated with only mild symptomology, but the demyelination became extensive in the vehicle-treated mice at days 21 (13.3±7.59% of the WM) and 28 (12.9±6.05%), whereas little additional demyelination was observed in the nicotine-treated EAE mice (5.1±3.07% on day 21 or 2.9±4.40 on day 28) (Fig. 3C).

**Figure 4:**
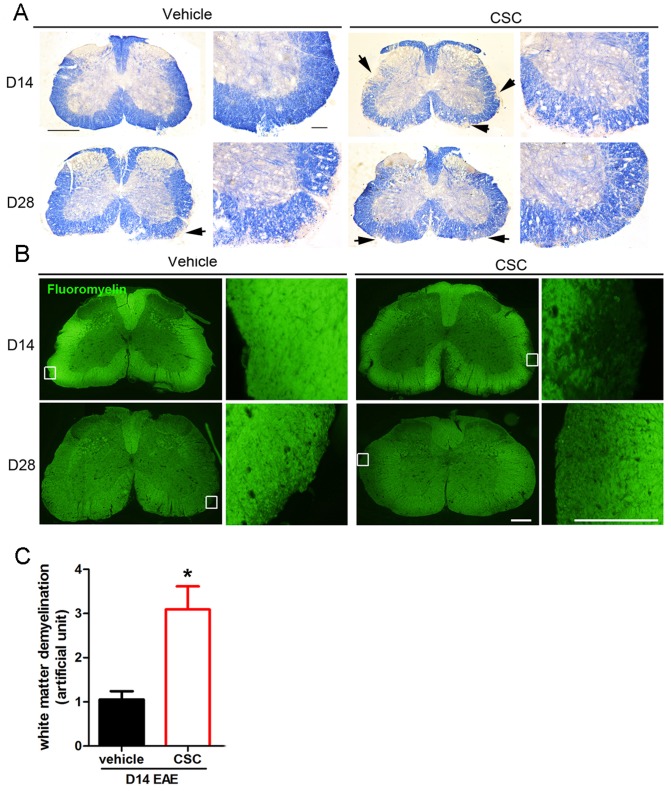
Increased demyelination in CSC-treated mice at day 14. Frozen sections of spinal cords were isolated from vehicle (saline/DMSO) and CSC-treated mice at day 14 and day 28 of EAE. Eriochrome cyanine **(A)** or fluoromyelin **(B)** was used to visualize demyelination. Areas of demyelination are indicated by diminished fluorescence; boxed regions are shown at higher magnification. **(C)** Demyelinated areas from **(B)** were measured using ImageJ and calculated based on equation listed in Methods (n = 4, *p<0.05). The levels of demyelination at day 14 in saline- or nicotine-infused spinal cords are also shown for comparison.

In CSC-treated EAE samples, the demyelination at day 14 (6.33±0.83%), was three fold higher than the demyelination observed in control samples (2.16±0.4%, Fig. 4A, B). However, no significant difference in myelination was found between CSC- and vehicle-treated samples at day 28 (Fig. 4B). These results correlated with symptomology showed in clinical scores.

Taken together, our results indicate that the non-nicotine cigarette components in CSC have adverse effects on EAE severity, as evidenced by a higher clinical score and increased spinal cord demyelination. We also confirmed that nicotine does have protective effects on EAE outcomes, through reduced disease score, loss in body weight, and demyelination.

### Nicotine and non-nicotine components have opposite effects on microglial activation during early EAE

As immune cell function is an essential component of disease progression and resolution in EAE, the immunomodulatory functions of nicotine could underlie the nicotine-induced protective effects described thus far. Microglia express most of the nicotine receptors that have been linked to immune-regulatory functions [29].

**Figure 5:**
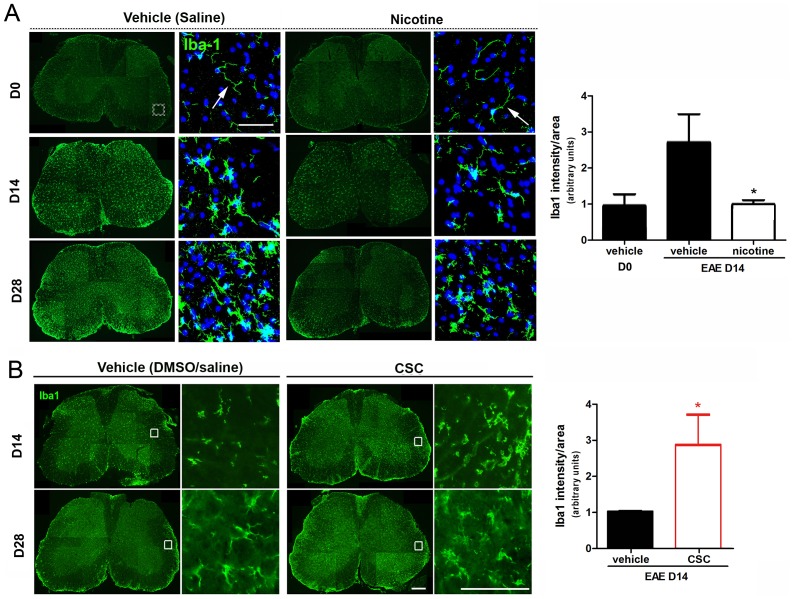
Effects of nicotine and non-nicotine components of cigarette smoke on microglia/macrophage activation during EAE. **(A)** Iba1 staining (green) was used to detect microglia in vehicle (saline), CSC-treated and nicotine-treated samples collected at D0, D14 and D28 during EAE. DAPI is indicated by blue fluorescence. Higher magnifications show morphologies of microglia in white matter (the box in D0 vehicle indicates the location of the high magnification images). **(B)** Iba1 staining (green) was used to detect microglia in vehicle (saline/DMSO) and CSC-treated samples collected at D14 and D28 during EAE. Quantification of Iba1 signals at days 0 and 14 of EAE after vehicle and nicotine (n = 3–4), or day14 CSC treatment (n = 4). Bar = 20 µm. *p<0.05.

We have previously reported that changing the timing or decreasing the extent of microglial activation affects the outcome of EAE [17]. To evaluate whether nicotine and non-nicotine components in cigarette smoke affect microglial activation, anti-Iba1 antibody was used to visualize microglial morphology in the experimental settings previously described. Microglia in spinal cord sections prepared from control animals on day 0 of the experimental protocol exhibited the ramified morphology characteristic of non-activated, “resting” microglia (arrow, Fig. 5A). By day 14 of the EAE induction protocol, large numbers of microglia were clustered at the periphery of the WM in spinal cords from vehicle-treated mice and they exhibited an activated morphology characterized by enlarged cell bodies and retracted cell processes. However, CSC infusion into the spinal cord resulted in extensive peripheral clustering and widespread microglial activation and differentiation into amoeboid morphology (Fig. 5B), which characterized the highest levels of activation. In contrast, peripheral WM clustering of microglia was not observed in spinal cords sections prepared from the EAE mice treated with nicotine at this time point, and the microglia observed were predominantly still ramified, or had undergone only partial activation. By day 28 of EAE, microglia in both the vehicle and nicotine-treated spinal cord sections had begun to reverse their activation process while microglia in CSC-treated samples still remained activated.

Quantification of the anti-Iba1 staining intensity as a measure of microglial/macrophage number and activation revealed an increase in the EAE vehicle-treated sections at day 14 of the protocol but no increase in the nicotine-treated sections (quantifications in the graph). In contrast, Iba1+ immunofluorescence was almost 3 fold higher in CSC-treated animals at day 14 than in control sections (quantification in the graph), either due to increased microglial activation/proliferation or macrophage infiltration. These results demonstrate that non-nicotine components of cigarette smoke exacerbate microglial/macrophage activation at early time points of EAE while nicotine attenuates their activation.

### Nicotine and non-nicotine components have opposing effects on the M1 microglial subpopulation in the white matter at early stages in EAE

Resting microglia differentiate into pro-inflammatory M1 microglia starting at the onset of EAE and remain detectable throughout the course of disease [16]. Differentiation of resting microglia into anti-inflammatory M2 microglia occurs less quickly and less efficiently, resulting in a predominance of M1 microglia and a pro-inflammatory milieu [15], [30]. We showed above that microglial activation during EAE is stimulated by non-nicotine components in CSC, but suppressed by nicotine administration; here we address whether these distinct effects of nicotine and non-nicotine components of cigarette smoke are due to the different extent of their effect on microglial subtypes.

**Figure 6:**
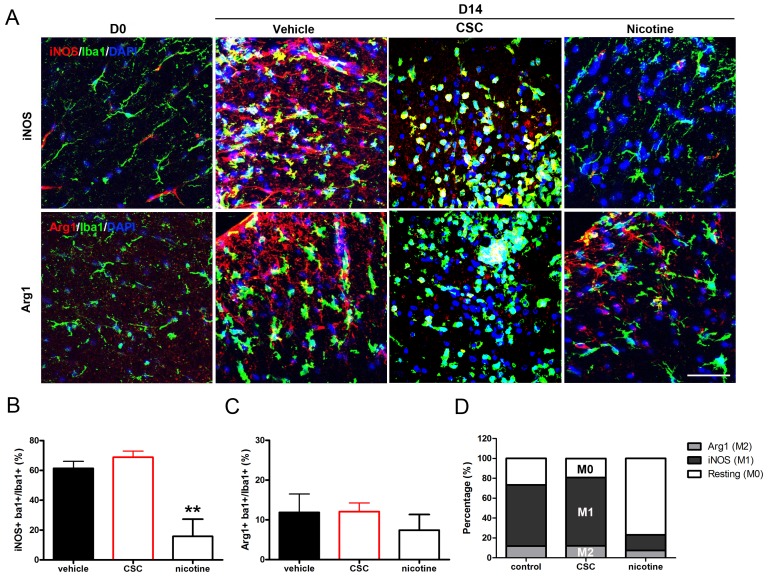
Non-nicotine components of cigarette smoke and nicotine have opposite effects on microglial state during early stages of EAE. (**A**) Resting Iba1+ (M0), pro-inflammatory (M1) and anti-inflammatory (M2) microglia were identified by labeling with anti-iNOS/Iba1 and anti-Arg1/Iba1, respectively. iNOS^+/^Iba1^+^ (**B**) and Arg1^+/^Iba1^+^ (**C**) cells were counted and compared between vehicle (pooled DMSO/saline samples) (n = 4) and CSC (n = 3)/nicotine-treated (n = 3) samples. Percentages of each population after treatment are indicated in (**D**). Bar = 50 µm (**p<0.01).

Microglia in the spinal cord white matter were generally found to express low levels of both M1 (iNOS) and M2 (Arg1) markers in the absence of disease; these cells were defined as M0 resting microglia (Fig. 6A). A small number of microglia with a predominantly resting phenotype expressed iNOS at high levels, but none were observed to express Arg1. Induction of EAE and microglial activation resulted in substantial increases in both M1 and M2 microglia (Fig. 6A, day 14 Vehicle), but the majority were of an M1 phenotype. ∼73.2% of the microglia were activated at day 14 (Fig. 6D). Of these, 61.4±9.5% were iNOS+ (Fig. 6B) and 11.9±9.2% were Arg1+ (Fig. 6C).

CSC treatment did not significantly modify the ratios of iNOS+ M1 microglia (68.8±7.2, Fig. 6A, B) to Arg1+ M2 cells (12.1±3.8, Fig. 6A, C). In total, there was an increase in microglial activation after CSC treatment (80.9%, Fig. 6D, 5C). In contrast, only ∼23.2% of the microglia in nicotine-treated samples became activated (Fig. 6A, D), primarily due to a reduction in M1 microglia (15.8±20.0%, Fig. 6B). There was also a decrease in M2 microglia after nicotine treatment (Fig. 6C); however, the effect on the M2 microglia was smaller and not significant.

Therefore, both nicotine and non-nicotine components in cigarette smoke regulate microglial activation by affecting the M1 microglial/macrophage subpopulation. CSC slightly enhances the M1 markers assessed, which leads to a pro-inflammatory environment in the CNS and a faster disease progression. In contrast, nicotine application significantly suppresses the M1 markers examined, including MHCII and CD86 (data not shown), decreasing the M1 predominance, presumably creating a relatively anti-inflammatory environment and outcome.

### Non-nicotine components in CSC have cytotoxic effects on microglia

**Figure 7:**
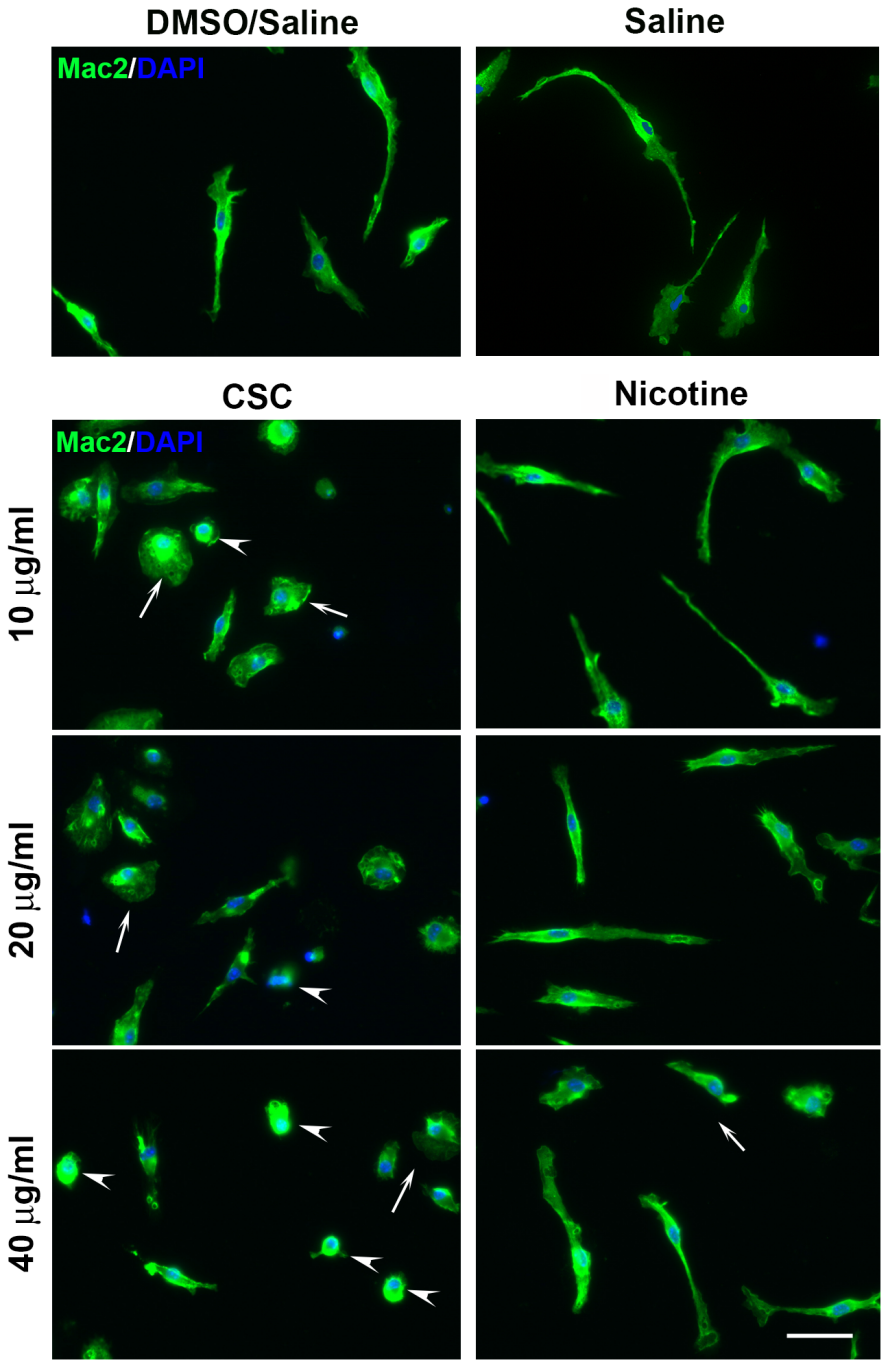
CSC exposure induces ameboid morphology in microglia. Primary microglia were treated with different concentrations of CSC and nicotine for 24 hours and cell morphology visualized by staining with Mac2 antibody. Arrows point to enlarged cell bodies and retracted cell processes. Arrowheads point to cell shrinkage after CSC treatment. (Bar = 20 µm, n = 4).

**Figure 8:**
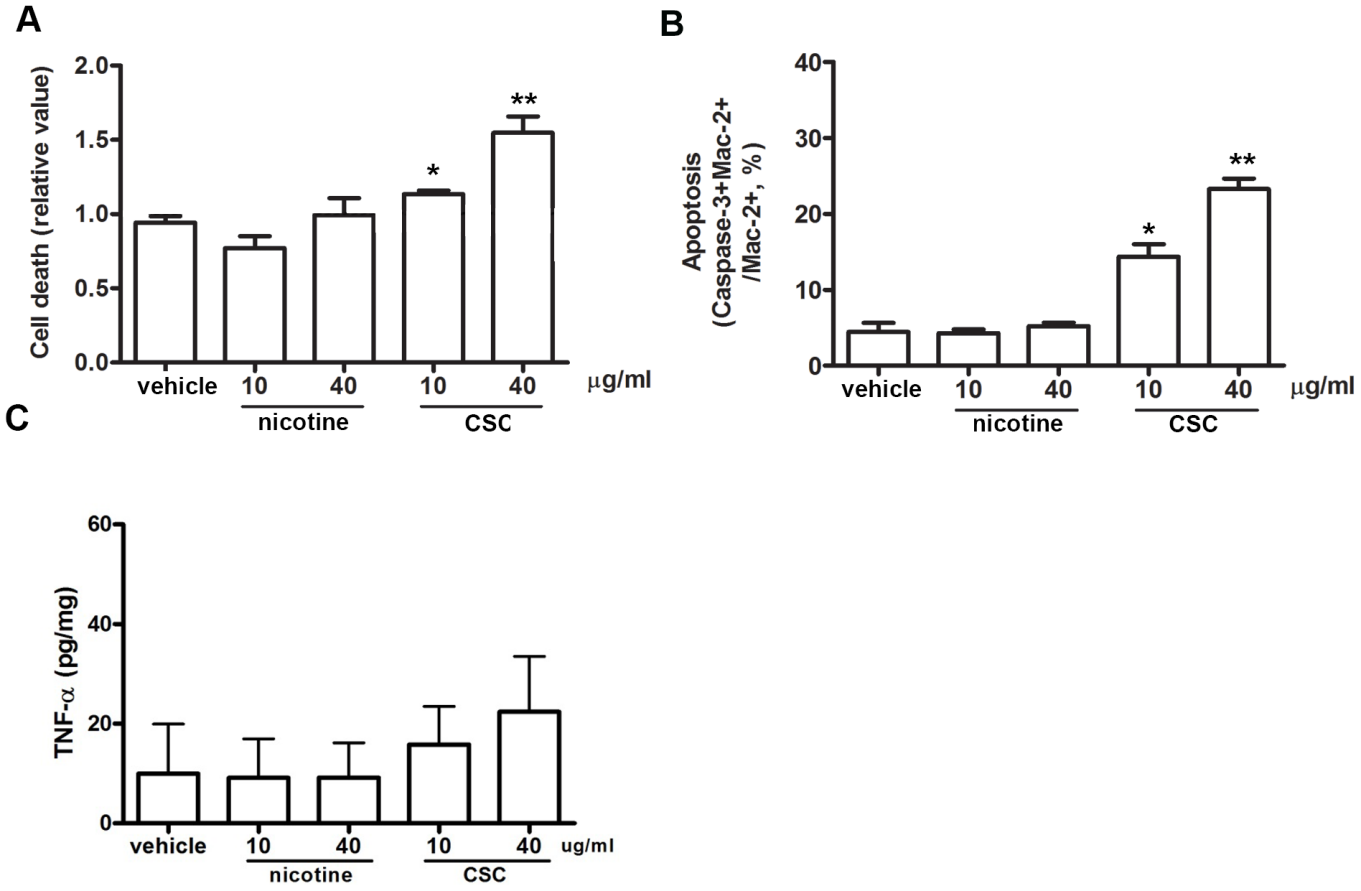
CSC exposure induces microglia apoptosis. Primary microglia were treated with the indicated concentrations of CSC or nicotine for 24 hours. **(A)** Media were collected and cell death was measured with a live/dead assay kit according to the manufacturer's instruction; (n = 4–7). Vehicle used was DMSO/saline. **(B)** apoptosis quantified through double staining with anti-Mac2 and anti-caspase-3. **(C)** TNF-α levels measured by ELISA as described in Methods. (n = 3; **p<0.01; *p<0.05 compared to vehicle).

To extend the findings above, we examined the effect of CSC on microglial activation and morphology *in culture*. At all concentrations used, CSC induced the microglia to change from a ramified morphology to the amoeboid state (arrows), while the same concentration of nicotine (approximately 40-fold greater than the nicotine found in CSC) did not have any effect (Fig. 7). Shrunken cell bodies were observed after CSC treatment (arrowheads, Fig. 7), suggesting that CSC might potentially be cytotoxic for microglia. This was found to be the case, as total cell death for microglia was increased with exposure to CSC but not nicotine (Fig. 8A), and this finding was even more dramatic for apoptosis, which increased more than 5-fold in the presence of CSC (Fig. 8B). These results were surprising, as *in vivo* administration of CSC promoted an increase in microglial activation (Fig. 5) and M1 polarization (Fig. 6). This could potentially be attributed to the fact that *in vitro* the CSC is acting exclusively on microglial cells, in the absence of other cell types. On the other hand, in the animal there are many more targets for CSC as well as a variety of interactions between microglia and other CNS cells, most likely astrocytes, which may be able to neutralize CSC-induced oxidative death. The drastic difference in microenvironment may be able to explain the difference between these experiments. The concentration of nicotine in CSC we applied was ∼2–3%. To examine whether this low level of nicotine in CSC may contribute to any cytotoxic effects, nicotine (0.3 µg/ml nicotine for 10 µg/ml CSC and 1.2 µg/ml nicotine for 40 µg/ml CSC) was added to the culture medium of primary microglia. Cell viability was not affected by nicotine (Figure S3), suggesting that non-nicotine components in CSC led to death of microglia. CSC showed a trend towards higher TNF levels released from the microglia, but this increase did not reach significance (Fig. 8C). Taken together, these findings again suggest that the non-nicotine components in CSC are responsible for the detrimental effects of cigarette smoke on microglial activation/function and EAE outcome.

### Acrolein contributes to the cytotoxic effects of CSC on microglia

**Figure 9:**
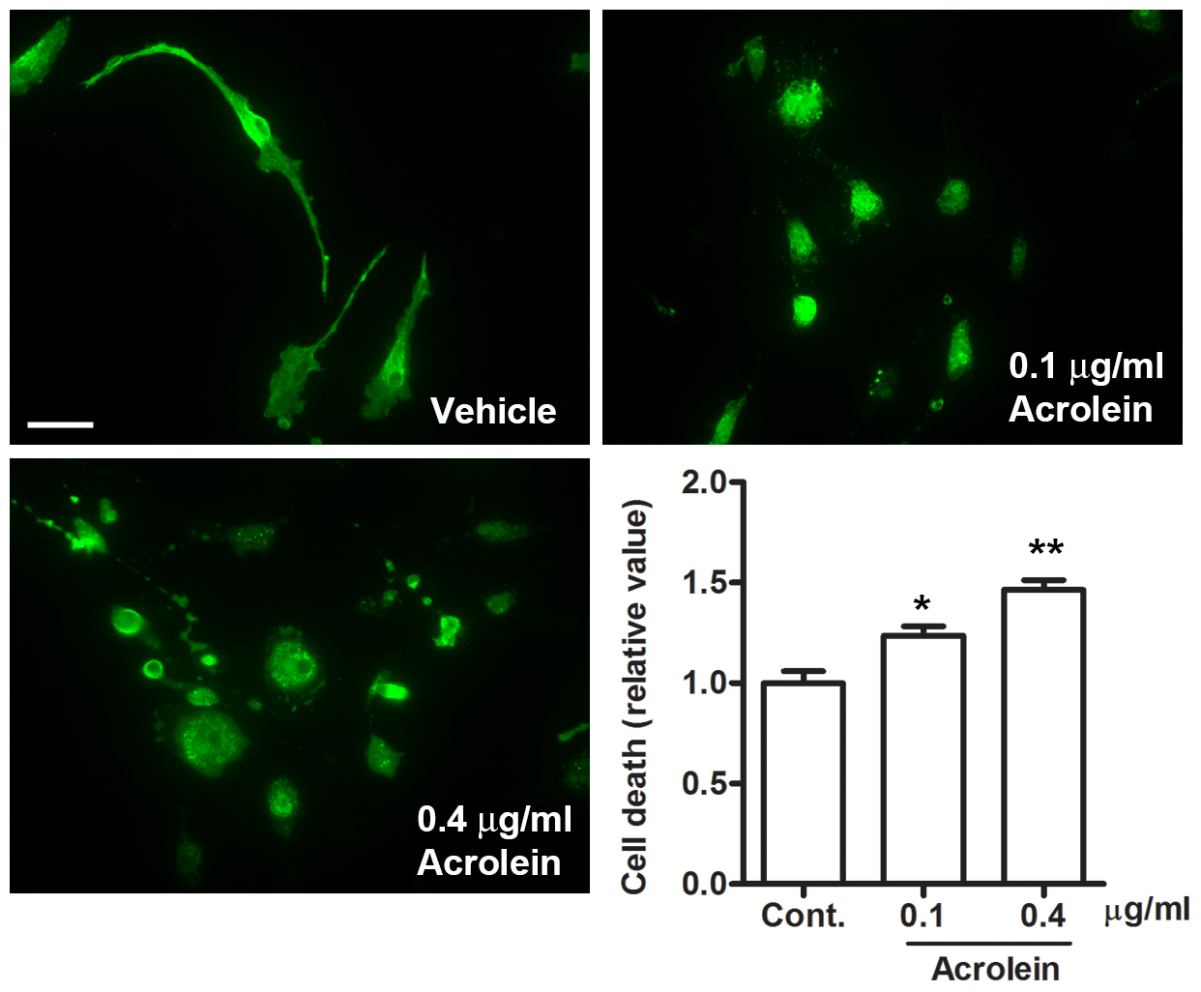
Acrolein induces cell death. Primary microglia were treated with acrolein for 24 hours and stained with Mac2 antibody. Cell death was quantified using a live/dead assay. Bar = 20 µm (n = 3; **p<0.01; *p<0.05).

Candidate compounds potentially responsible for the cytotoxic effects of CSC were explored. Acrolein, a volatile component of cigarette smoke, is a product of oxidative stress and lipid peroxidation, and increased expression of acrolein-lysine is found in EAE samples [31]. Inhibition of acrolein by hydralazine (an acrolein scavenger) has been reported to significantly improve behavioral outcomes for EAE [31]. We applied acrolein to microglia *in culture* to examine whether it could recapitulate the cytotoxic effects of CSC. Concentrations higher than 1 µg/ml killed all of cells (data not shown). Being treated with acrolein even at lower concentrations (0.1 µg/ml and 0.4 µg/ml) for 24 hours sufficed to elicit cell death to a significant extent (Fig. 9). These concentrations of acrolein are equal or lower than that contained in the CSC used in Fig 7. We calculated the concentration of acrolein as 0.18 µg/ml or 3.2 µM in the 40 µg/ml CSC (0.091 µg/ml in the 20 µg/ml and 0.045 µg/ml in the 10 µg/ml CSC). The calculation was based on the amount of total particulate matter (TPM) per 3R4F cigarette (which is 11 mg), and the amount of acrolein per cigarette (approximately 50 µg) [32]–[35]. Further, we observed that CSC elicited cell death in microglia at even higher levels than acrolein alone. However, when acrolein in CSC was inhibited through treatment with hydralazine, as previously described [36], [37], this effect was significantly attenuated, although not completely reduced to control levels (Figure S4). Thus, our findings suggest that acrolein may, at least in part, mediate the cytotoxic effects of CSC. As acrolein can promote oxidative microglial cell death *in vitro*, we evaluated whether such oxidative damage also occurred *in vivo*. Spinal cords from mice treated with CSC or DMSO control were stained for nitrotyrosine, an indicator of oxidative stress. There was notable induction of nitrotyrosine in CSC-infused animals compared to controls, which suggests that acrolein has a similar mechanism during EAE (Figure S5). Taken together, these results provide one mechanism to potentially rationalize how CSC aggravates EAE, in particular in the earlier phases of the disease.

### Nicotine has an anti-inflammatory function under MS/EAE relevant conditions in culture

**Figure 10:**
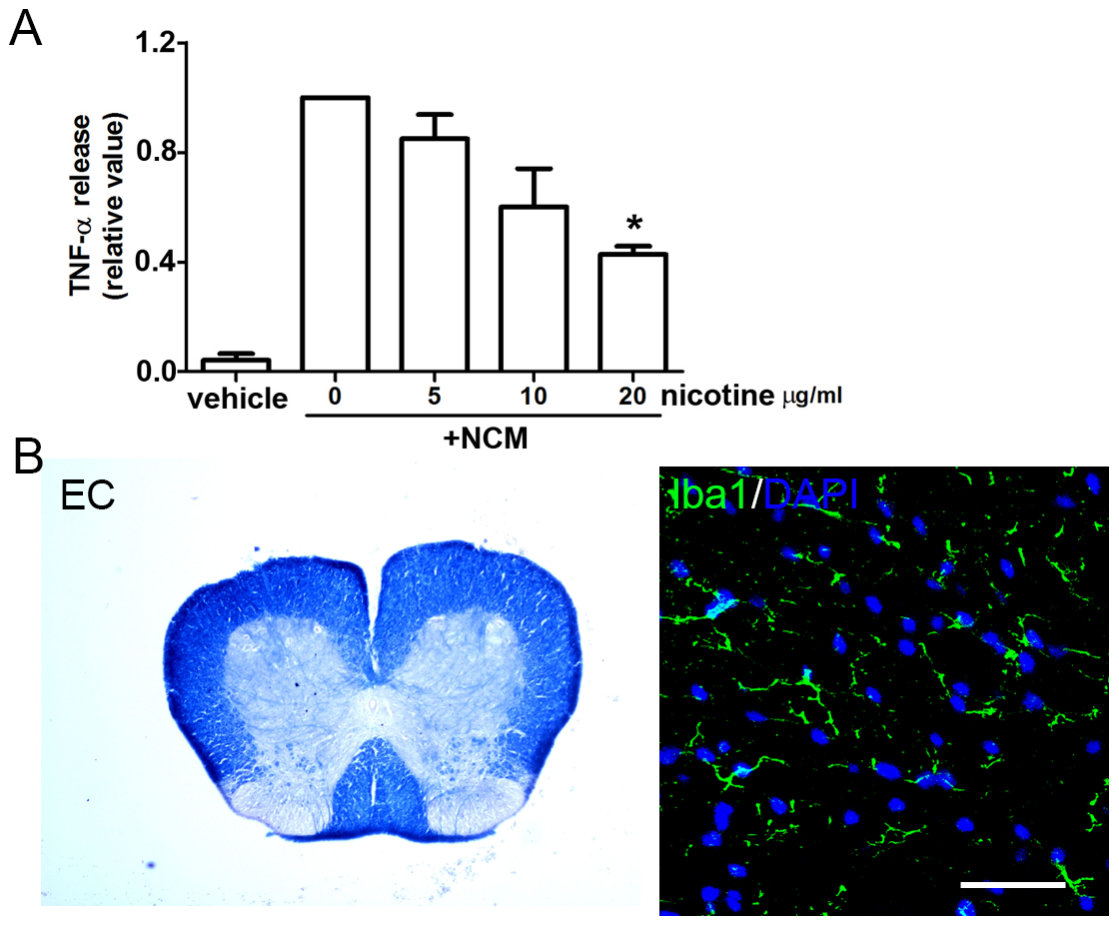
Nicotine has an anti-inflammatory function under MS/EAE relevant conditions in culture. **(A)** Primary microglia were exposed to nicotine for 30 minutes and then treated with conditioned medium from glutamate-stimulated neurons (NCM) for 24 hours. The media from the treated microglia were collected and TNF-α levels measured by ELISA as described in Methods (n = 4). **(B)** Frozen sections of spinal cords were isolated from mice treated with nicotine (28 days, 200 mg/ml nicotine ditartrate). EC staining was applied to visualize myelination in spinal cord sections. Inflammation was evaluated by staining of Iba1, CD45, Arg1 and iNOS. Staining of caspase-3 was used to visualize cell death. The results were compared with those from vehicle-injected mice. Bar = 50 µm (n = 3–4; *p<0.05).

The effect of nicotine on microglial activation was further examined using cell cultures. Glutamate excitotoxicity-induced neurodegeneration has been described in MS and observed in the EAE model [38], [39]. To mimic the microglial microenvironment in MS/EAE, we treated primary microglia with neuronal conditioned medium (NCM) and measured the level of pro-inflammatory cytokine TNF-α in the microglial medium. As shown in Fig. 10A, nicotine supplementation decreased TNF-α release from NCM-stimulated microglia and the nicotine effect was dose-dependent, confirming that nicotine has an anti-inflammatory function under MS/EAE relevant conditions. Nicotine alone did not induce demyelination, inflammation or cell death, *in vivo* (Fig. 10B) or *in vitro* (Fig. 8).

### Therapeutic nicotine administration attenuates EAE symptoms

**Figure 11:**
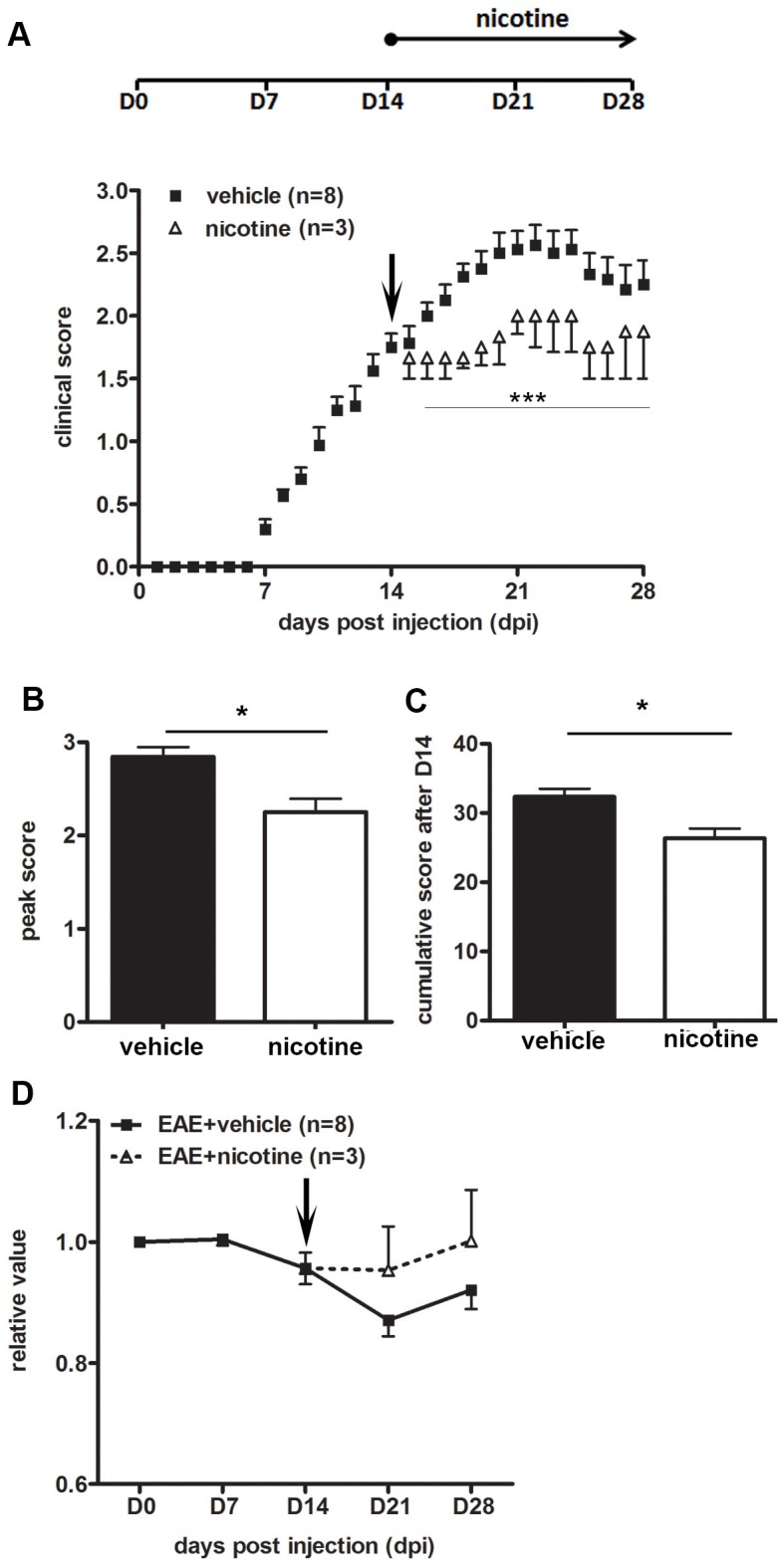
Therapeutic administration of nicotine attenuates EAE symptoms. **(A)** 14-day pumps filled with 200 mg/ml nicotine were placed in the backs of EAE mice at day 14 (***<0.001). Peak score **(B)** as well as cumulative score **(C)** from day 14 to day 28 were recorded and compared between the vehicle (n = 8) and nicotine-treated (n = 3) groups. **(D)** Weekly weights were plotted as a percentage of day 0 weights. (***p<0.001; *p<0.05)

Since nicotine confers benefits in EAE when provided during the initiation phase, we next assessed the possibility of using nicotine therapeutically, i.e., after the mice become symptomatic. Nicotine pumps were implanted in the backs of EAE mice at day 14, at which point the mice had developed mild clinical signs of disease (Fig. 11A). Although nicotine was not provided until after EAE initiation, when the mice were symptomatic, it still had a beneficial effect in that the symptom progression was halted (peak score of 2.25±0.25 as opposed to 2.80±0.30 for the vehicle-infused mice, Fig. 11A, B). Cumulative scores (32.3±3.3 versus 26.3±2.4, Fig. 11C) and body weights (Fig. 11D) were similarly different between the control and treatment groups. Therefore, nicotine could potentially be used as a therapeutic option if its utility in EAE is predictive for MS.

## Discussion

It is well accepted that cigarette smoking increases susceptibility to MS and worsens the disease [1], [40]. However, definition of the component(s) in cigarettes that mediate these effects has not been undertaken. In this study, we assessed the effects of nicotine and non-nicotine components of cigarette smoke on EAE. We found that nicotine significantly ameliorated EAE symptoms, including clinical score, demyelination, and microglial activation. In contrast, non-nicotine components worsened the disease severity at early stages of disease, including disease onset, demyelination and microglial activation, consistent with results from previous epidemiological studies showing that MS progressed faster in patients who smoked [4]. Our results suggest that the non-nicotine components could be responsible for the detrimental effects of cigarette smoking on MS, whereas nicotine may confer benefit.

Multiple factors could possibly contribute to nicotine's positive effects on EAE/MS, since nAChRs are found expressed on different cell populations, including neurons, microglia/macrophages [9], T cells [41], dendritic cells [10], and even oligodendrocyte progenitors [42] and endothelial cells [43]. Type 1 T helper cells (Th1) and Type 17 T helper cells (Th17) are the main pathogenic cells of EAE. Anti-inflammatory Th2 and regulatory T cells (Treg) normally protect against autoimmunity by inducing tolerance of self-antigens, however, their functions are impaired during the early stages of disease [44]. Nicotine has been shown to suppress the reactivity of pro-inflammatory T helper cells (Th1) and increase that of anti-inflammatory Th2 and immunosuppressive Treg cells in the CNS during EAE [11]. Moreover, as shown by Nizri *et al*., *in vitro* nicotine exposure reduces production of Th1 and Th17 cytokines and induces a shift towards Th2 in response to an encephalitogenic antigen [45].

In this study, we focused on nicotine's effect on microglial functions during EAE. We previously demonstrated that changing the timing and extent of activation [17] or the differentiation path [22] of microglia changed the outcomes of EAE. Here, we examined nicotine's effect on microglia in the context of these states and found that nicotine significantly delayed and inhibited microglial activation during EAE, and that this inhibition primarily proceeded through suppression of M1 (iNOS^+^) microglial differentiation. In culture, nicotine decreased TNF-α release from NCM-stimulated microglia. Previous studies demonstrated that M1 microglia promote T cell differentiation toward Th1 and Th17 fates [46], induce neurodegeneration [38] and impair remyelination [47]. These effects of M1 microglia were mediated primarily by TNF-α [38], [46], [48], [49]. Therefore, biased suppression of M1 microglia through nicotine administration supports an anti-inflammatory environment, possibly re-balancing T cell populations, protecting neurons and myelin sheaths from insults, and promoting recovery of the tissues.

It was reported that nicotine inhibited microglia/macrophage activation primarily through binding to α7nAChRs [50], [51]. Nevertheless, attenuation of EAE outcomes by nicotine was only partially reversed in α7-/- mice, which suggested that other nAChRs subunits might also contribute to the impact of nicotine on EAE [29]. Ohnishi *et al*. showed that, besides α7, β2-containing nAChRs were involved in nicotine's effects on thrombin-activated microglia [52]. In addition, α5 subunits contributed to immune regulatory functions, because the absence of α5 increased severity of experimental colitis [53]. Taken together, nicotine could potentially regulate microglia functions and EAE outcomes through targeting multiple nAChRs pathways.

In contrast, we demonstrated that CSC had a detrimental effect on EAE progression. However, the significant effect was only achieved during early stages of EAE. This might be due to the fact that the inflammation in mice due to ongoing EAE is more severe than the inflammatory effects mediated by this concentration of CSC. The highest concentration of CSC that we could use *in vivo* was 20 mg/ml, which contains only 2–3% of nicotine. If we had been able to deliver significantly higher concentrations of CSC, then more severe EAE symptoms might have been encountered. Nonetheless, we used CSC-treated EAE samples and cell culture to explore how CSC affects inflammation. Our results indicate that CSC both activates microglia, and eventually becomes cytotoxic to the cells. Other studies have shown that low concentrations of CSC induce inflammatory cytokine release from pulmonary macrophages, while higher concentrations cause cell death [54]. These reports support our findings that CSC enhances inflammation in EAE mice. We and others have previously shown that inhibition of microglia/macrophage function protects from EAE (e.g., Bhasin et al., 2007), further suggesting that CSC adversely affects EAE scores. Further, we have shown that inhibition of acrolein in CSC by hydralazine significantly reduces cell death in treated primary microglia. However, this effect does not fully rescue this phenotype, as there is still significantly more cell death compared to controls. This suggests that acrolein could mediate part, but not all, of CSC's negative effects *in vitro* and *in vivo*.

As discussed previously, M1 microglia have detrimental effects during MS/EAE. However, it does not mean that pro-inflammatory mediators like M1 microglia and Th1 cells only contribute adversely to MS/EAE. Controlled microglial activation and T cell infiltration promote optic nerve recovery from injury [55]. Appropriate amounts of M1 differentiation have been shown to promote neurogenesis and oligogenesis, whereas excessive activation is inhibitory [48], [49]. Rebalancing the pro-inflammatory and anti-inflammatory factors is critical for disease progression and recovery, which is supported by our results showing the opposite courses of disease development of CSC- and nicotine-treated EAE animals. Results from our lab and others suggest that nicotine could be a good candidate as an inflammatory regulator since it suppresses pro-inflammatory differentiation of microglia and T cells [45] without affecting cell viability. We also demonstrated that nicotine administration significantly limits exacerbation and disease severity in established EAE. This is the first study that provides evidence to support the proposal that nicotine could be used as a therapy for on-going MS. The therapeutic effect of nicotine transdermal patches on MS/EAE is currently under investigation. However, some considerations may apply to the proposal to use nicotine. Although nicotine ameliorated symptoms of EAE in our study here, it has been associated with inflammation in the respiratory system and cancer [56]–[58]. The potential side effects of nicotine patches will need to be evaluated carefully.

Recent studies by Odoardi *et al*. have demonstrated that the lung contributes to the T cell activation and migration that are required for MS/EAE initiation [59]. This may explain why lung infections are associated with MS progression. Importantly, there are many components in cigarette smoke that can modify the physiology of the lung but have not yet been shown to have direct effects on the CNS. It is possible that one of the pathways through which CSC affects EAE is through changing the inflammatory environment in the lungs and thus altering T cell differentiation. However, the respiratory system was not included in our study because we used osmotic pumps to deliver the CSC systemically into mice and thus the CSC components did not directly interact with the respiratory system. As this method is not an accurate representation of the delivery of cigarette smoke in human MS patients, to better model the effects of CSC on MS/EAE, a method that subjects mice directly exposed to cigarette smoke, such as an inhalation chamber [60], may need to be employed.

## Supporting Information

Figure S1 **EAE scores using saline or saline/DMSO vehicles.** EAE was induced by injection of MOG_35–55_ in CFA and pertussis toxin. Saline or DMSO in saline (50%) was infused into EAE mice starting at day 0 of EAE for 28 days. Peak score and cumulative score were compared.
(TIF)

Figure S2 **Lower concentrations of nicotine do not have significant effects on EAE.** EAE was induced by injection of MOG_35–55_ in CFA and pertussis toxin. Nicotine (10 mg/ml) was infused into the mice starting at Day 0, with saline as vehicle (n = 3 for each treatment).
(TIF)

Figure S3 **Nicotine exposure does not affect microglial death.** Primary microglia were treated with the indicated concentrations of nicotine for 24 hours. Media were collected and cell death was measured with a live/dead assay kit according to the manufacturer's instruction (n = 4).
(TIF)

Figure S4 **Hydralazine reverses CSC-mediated cell death.** Primary microglia were treated with 450 nM hydralazine, 40 µg/ml CSC, or both for 24 hours, with DMSO for control. Media were collected and cell death was measured with a live/dead assay kit according to the manufacturer's instruction (n = 3, **p<0.01; *p<0.05).
(TIF)

Figure S5 **CSC induces oxidative stress in mouse spinal cords during EAE.** Spinal cord sections from D0 control and DMSO or CSC-infused animals on D14 and D28 post EAE induction were stained for nitrotyrosine (green), a marker for oxidative stress, or DAPI (blue). Bar = 50 µm.
(TIF)
