# Supplementary figures and images for: The Experimental Autoimmune Encephalomyelitis Disease Course Is Modulated by Nicotine and Other Cigarette Smoke Components
Source: PLoS One. 2014 Sep 24;9(9):e107979. doi: 10.1371/journal.pone.0107979 (PMC4176721; doi:10.1371/journal.pone.0107979)

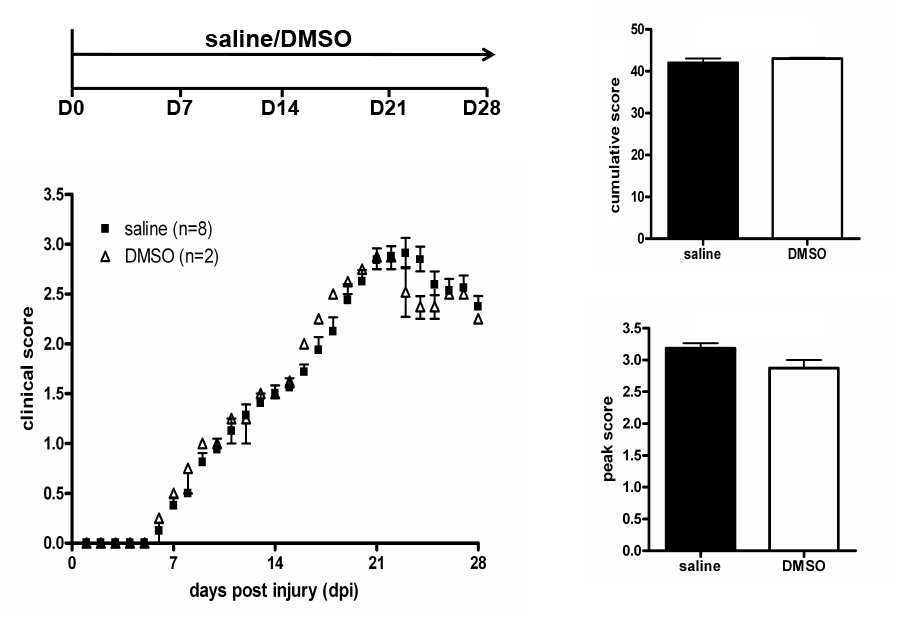

Supplement: Figure S1 — EAE scores using saline or saline/DMSO vehicles. EAE was induced by injection of MOG35–55 in CFA and pertussis toxin. Saline or DMSO in saline (50%) was infused into EAE mice starting at day 0 of EAE for 28 days. Peak score and cumulative score were compared. (TIF) [file pone.0107979.s001.tif]

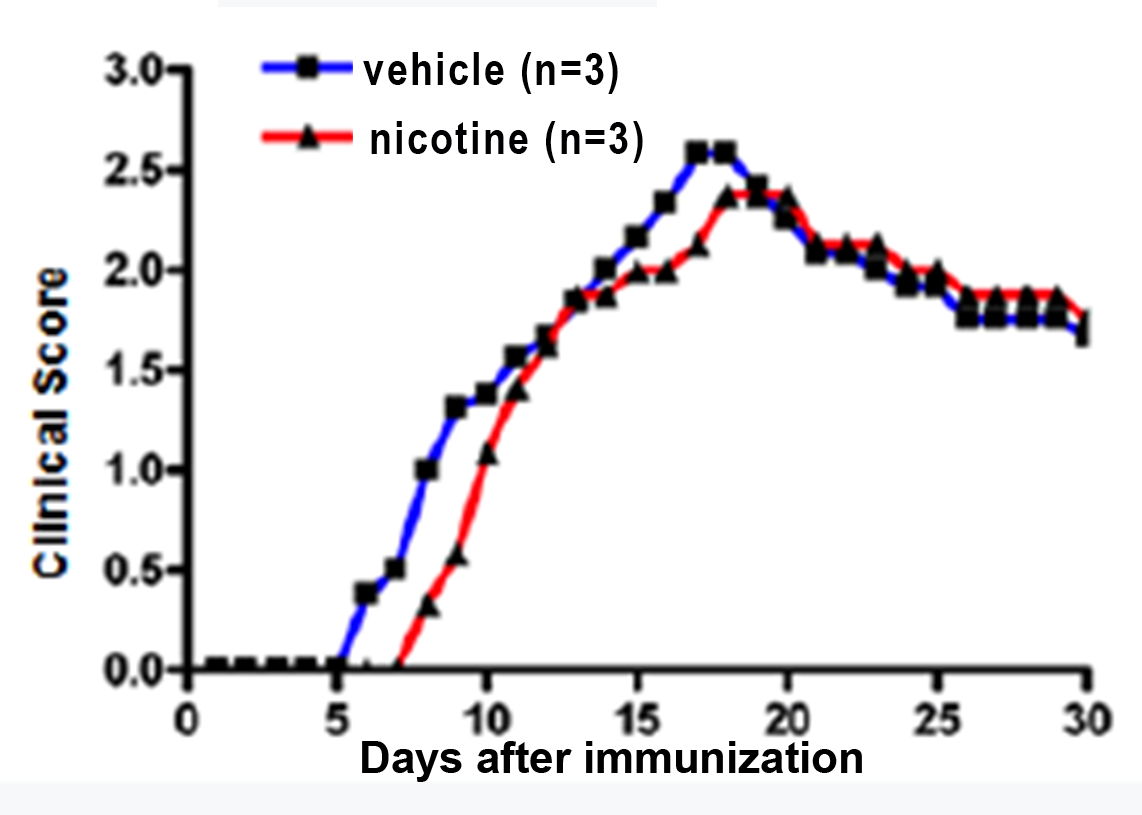

Supplement: Figure S2 — Lower concentrations of nicotine do not have significant effects on EAE. EAE was induced by injection of MOG35–55 in CFA and pertussis toxin. Nicotine (10 mg/ml) was infused into the mice starting at Day 0, with saline as vehicle (n = 3 for each treatment). (TIF) [file pone.0107979.s002.tif]

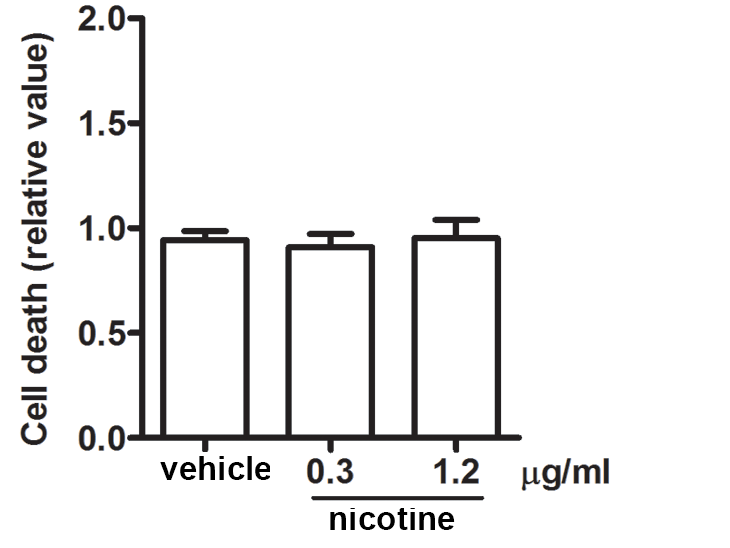

Supplement: Figure S3 — Nicotine exposure does not affect microglial death. Primary microglia were treated with the indicated concentrations of nicotine for 24 hours. Media were collected and cell death was measured with a live/dead assay kit according to the manufacturer's instruction (n = 4). (TIF) [file pone.0107979.s003.tif]

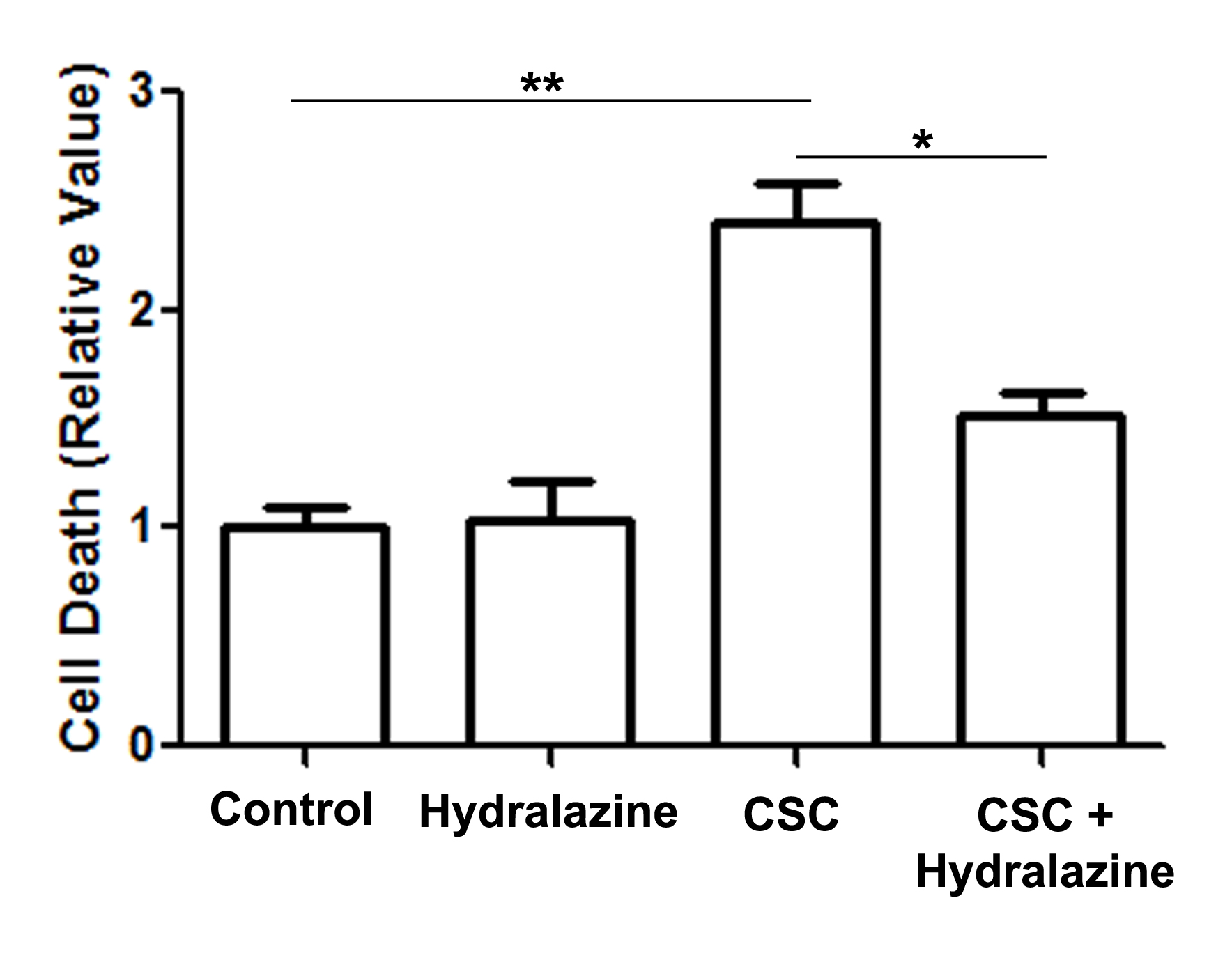

Supplement: Figure S4 — Hydralazine reverses CSC-mediated cell death. Primary microglia were treated with 450 nM hydralazine, 40 µg/ml CSC, or both for 24 hours, with DMSO for control. Media were collected and cell death was measured with a live/dead assay kit according to the manufacturer's instruction (n = 3, **p<0.01; *p<0.05). (TIF) [file pone.0107979.s004.tif]

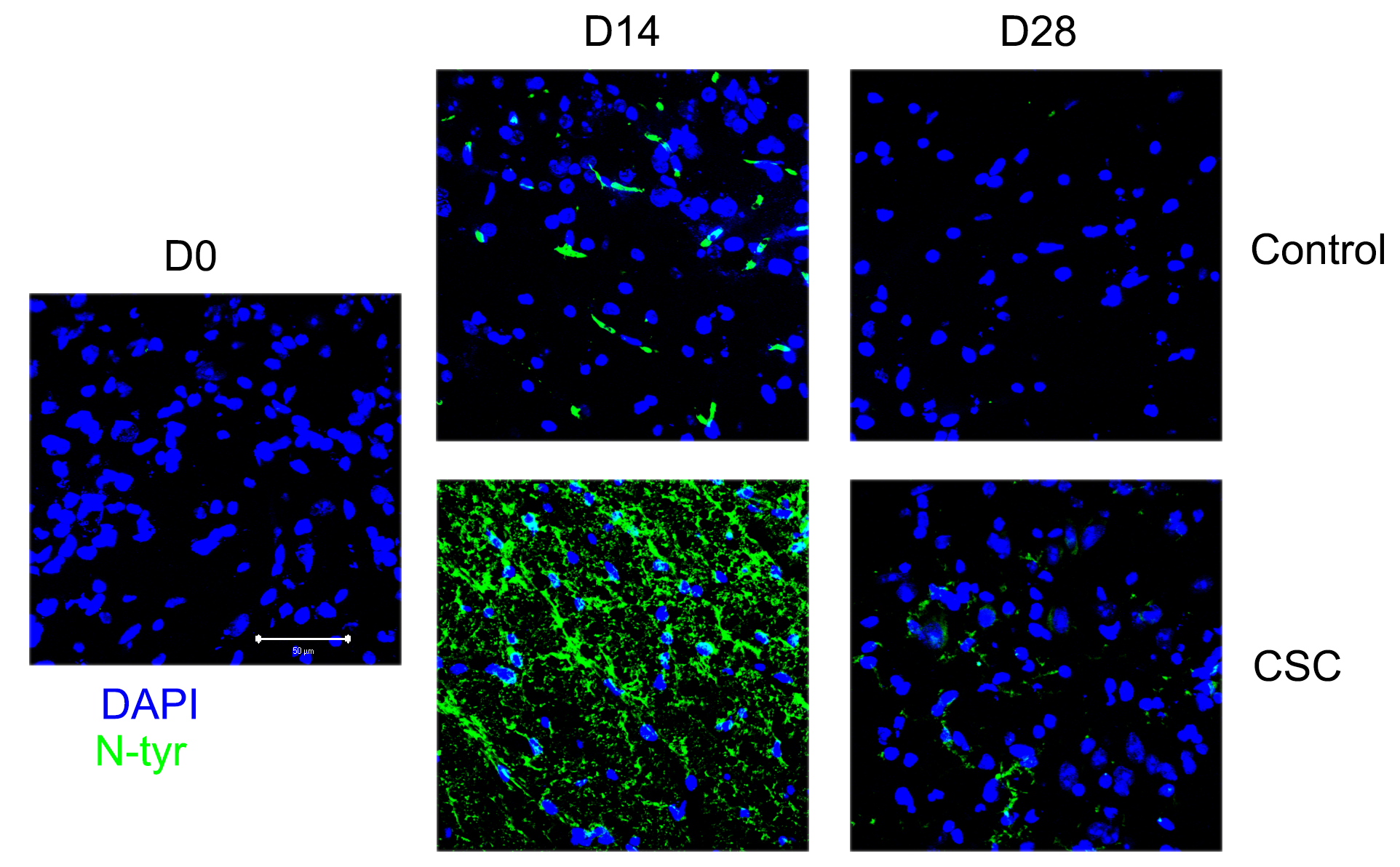

Supplement: Figure S5 — CSC induces oxidative stress in mouse spinal cords during EAE. Spinal cord sections from D0 control and DMSO or CSC-infused animals on D14 and D28 post EAE induction were stained for nitrotyrosine (green), a marker for oxidative stress, or DAPI (blue). Bar = 50 µm. (TIF) [file pone.0107979.s005.tif]
